# Promoting Anti-Racism in Clinical Practice: Lessons Learned in the Process of Removing the Race Coefficient from the Estimated Glomerular Filtration Rate Algorithm

**DOI:** 10.1089/heq.2023.0095

**Published:** 2023-11-30

**Authors:** Carla Boutin-Foster, Camille A. Clare, Jameela Yusuff, Moro Salifu

**Affiliations:** ^1^ Department of Medicine, SUNY Downstate Health Sciences University College of Medicine, Brooklyn, New York, USA.; ^2^ Department of Obstetrics and Gynecology, SUNY Downstate Health Sciences University College of Medicine, Brooklyn, New York, USA.

**Keywords:** anti-racism, clinical practice, medicine, race based clinical algorithms

## Abstract

**Background:**

Promoting anti-racism in medicine entails naming racism as a contributor to health inequities and being intentional about changing race-based practices in health care. Unscientific assumptions about race have led to the proliferation of race-based coefficients in clinical algorithms. Identifying and eliminating this practice is a critical step to promoting anti-racism in health care. The New York City Department of Health and Mental Hygiene (NYC-DOHMH) formed the Coalition to End Racism in Clinical Algorithms (CERCA), a health system consortium charged with eliminating clinical practices and policies that perpetuate racism.

**Objective:**

This article describes the process by which an academic medical center guided by the NYC-DOHMH tackled race-based clinical algorithms.

**Methods:**

Multiple key interested parties representing department chairs, hospital leaders, researchers, legal experts, and clinical pathologists were convened. A series of steps ensued, including selecting a specific clinical algorithm to address, conducting key informant interviews, reviewing relevant literature, reviewing clinical data, and identifying alternative and valid algorithms.

**Key Outcomes:**

Given the disproportionately higher rates of chronic kidney disease risk factors, estimated glomerular filtration rate (eGFR) was prioritized for change. Key informant interviews revealed concerns about the clinical impact that removing race from the equation would have on patients, potential legal implications, challenges of integrating revised algorithms in practice, and aligning this change in clinical practice with medical education. This collaborative process enabled us to tackle these concerns and successfully eliminate race as a coefficient in the eGFR algorithm.

**Conclusions:**

CERCA serves as a model for developing academic and public health department partnerships that advance health equity and promote anti-racism in practice. Lessons learned can be adapted to identify, review, and remove the use of race as a coefficient from other clinical guidelines.

## Background

### Historical context

Despite the consensus among scientists that race is a social construct that is devoid of biological basis, race is consistently misused as a marker of genetic variation in medicine.^1,2^ The concept of race has been used to subjugate people of African descent, confer a status of inferiority, and justify their enslavement. It is important to understand the genesis of racialized medicine and of race-based coefficients in clinical algorithms. One of the earliest clinical tests that demonstrates how race was intentionally used to devalue people of African descent and justify slavery is the spirometer. In his “Notes on the State of Virginia (circa 1780),” Thomas Jefferson remarked about differences in lung capacity between enslaved Black and White colonists.^3^

In this document, he describes what he presumed to be a “difference of structure in the pulmonary apparatus” of enslaved Black men. The misclassification of race as a biological entity continued into the 19th century as evidenced by diagnoses such as Drapetomania, first espoused by Dr. Samuel Cartwright (1851), which categorized the desire for enslaved Black people to run away and Dysaethesia Aethiopica (1851), an alleged mental health illness associated with “rascality” and partial insensitivity of the skin in response to pain when a person is subjected to punishment.^4^ Thomas Jefferson's hypothesis regarding differences in lung capacity between Black and White individuals provided fodder to Dr. Samuel Cartwright's unethical experimentation using spirometers. He interpreted his observation of an ∼20% difference in spirometry findings between Black enslaved people and White people as a “deficiency” in lung capacity of enslaved Black people. Relevant biological factors such as age, environmental factors, deplorable living, and working conditions were not considered. This belief was widely used to justify slavery because Black people were thought to be biologically deficient and forced labor was described as a way to “vitalize the blood.”^5^ This and similar misconceptions have become accepted in the modern day interpretation of pulmonary function, as well as other clinical tests.^6^

### Modern day practice

These false assumptions persist in modern day clinical practice and may contribute to the disparate ways in which pain is interpreted and treated based on the patient's race. Studies have found that Black patients are significantly less likely (57%) to receive analgesics for long-bone fractures compared to White patients (74%).^7^ Similarly, studies have found that Black children presenting with appendicitis are less likely to receive analgesia than White children with similar pain complaints.^8^ This differential threshold for acknowledgment and treatment of pain becomes evident early in medical education, as some medical students and residents have been found to hold false beliefs about the biological differences between Black and White patients.^9^

Using race as a biological modifier is problematic and exacerbates health disparities. Racial and ethnic differences in rates of cesarean births are well documented.^10^ When race is used in the estimation of successful vaginal birth after cesarean section (VBAC), it assigns a lower chance of successful vaginal birth for Black or Hispanic patients.^9^ This tool contributes to higher rates of cesarean birth and attended risks observed in Black and Hispanic patients. Moreover, when well-intentioned clinicians use race-based VBAC estimates to counsel their patients, they inadvertently perpetuate disparities because they offer different advice based on race or ethnicity and not biological data.^4^ The use of race in assessing risks of urinary tract infections (UTIs) in infants and toddlers assigns lower risks of UTIs for Black children, which contributes to missed diagnosis and missed opportunities for early treatment.^11^ The Fracture Risk Assessment Tool (FRAX) estimates lower 10-year risk of fracture for Black women due to osteoporosis, which contributes to a missed opportunity for prevention.^12^

### Examples of race-based clinical algorithms: estimated glomerular filtration rate

The Modification of Diet in Renal Disease (MDRD) Study equation and the Chronic Kidney Disease Epidemiology Collaboration (CKD-EPI) equation are the most widely used equations for estimating glomerular filtration rates.^13^ Both algorithms include a race coefficient that reports higher glomerular filtration rates for Black patients. These equations are based on the observations of differences in creatinine according to how patients were identified and interpreted to indicate biological differences. This observation was attributed to muscle mass based on the presumption of race-based differences and led to the addition of a coefficient to improve the model. This race modifier created an artifact that raises the glomerular filtration rate of Black patients, which may lead to the assumption of better renal function, thus, missing opportunities for identifying early kidney disease, delaying referral to nephrologists and organ transplant teams, and missed opportunities to avoid potentially nephrotoxic medications.^14^

The recognition that these algorithms are unscientific, unethical, and even rooted in racism has led to a groundswell of support for eliminating race from clinical algorithms. Such a change in mindset and clinical practice included multi-institutional and multiregional advocacy movements rooted in community and clinician responses. A summarized approach to these efforts has been curated by the Institute of Healing and Justice in Medicine, including emails, memos, podcasts, and other resources.^15^ In 2019, Eneanya et al. questioned the use of race in estimates of kidney function, noting the lack of transparency and restrictions in access to care, further highlighting race as a social construct.^16^ Among specific notable scholars, Professor Dorothy Roberts called for similar actions much earlier,^17^ and Professor Lundy Braun advocated for the same in the use of the race based spirometry measurements.^18^

In May 2020, advocacy efforts by students at the University of Washington School of Medicine led to the successful removal of race from the estimated glomerular filtration rate (eGFR) in their clinical practice.^19^ Drs. Monica Hahn, Stephen Richmond, Juliana Morris, and Vanessa Grubbs led this work at the Zuckerberg San Francisco General Hospital (SFGH) and the University of California-San Francisco (UCSF), which also discontinued the race-based reporting of eGFR due to a 700-signatory petition in June 28, 2020, urging them to abolish race-based eGFR and race-based medicine.^20^ The National Kidney Foundation (NKF) and the American Society of Nephrology (ASN) convened a Task Force to reassess the inclusion of race in eGFR in 2021. Finally, the impact of the voice of patients and trainees cannot be understated in their role as activists and advocates in this work.^21^

This collective activism of medical students served as a catalyst for several other institutions and national organizations to take a similar stance. The NKF and the ASN convened a Task Force to reassess the inclusion of race in eGFR. This momentum was further fueled and accelerated by the disproportionate toll of the coronavirus disease 2019 (COVID-19) pandemic on Black communities, which sparked public outrage and outcry to address racism in health care. This caused several health care institutions to reflect on practices that contribute to health inequities. This confluence of events served as an opportunity for our institution to not only take a vocal stance against structural racism but also to put in place intentional and strategic steps to dismantle structural racism in the practice of clinical medicine. This article describes the collaborative process by which a local health department partnered with an academic medical center to remove race-based coefficients from clinical algorithms.

## Methods

### Coalition to end racism in clinical algorithms

In November of 2021, under the leadership of the Chief Medical Officer of the New York City Department of Health and Mental Hygiene, the Coalition to End Racism in Clinical Algorithms (CERCA) was established. The Coalition was tasked with advancing equity in clinical care by ending the inclusion of race-based adjustments in clinical algorithms.^22^ An important premise underlying this initiative was anti-racism.

Anti-racism in health care entails first acknowledging racism as a fundamental driver of health inequities and then taking intentional steps toward changing racist practices, policies, and behaviors.^14,23,24^ The anti-racism framework provided a platform for hospitals and health systems in New York City in partnership with the New York City Department of Health and Mental Hygiene (NYC-DOHMH) to initiate honest and deliberate conversations about race and racism.

The State University of New York Downstate Health Sciences University (Downstate) joined the CERCA team. Downstate is located in Brooklyn, the largest borough in New York City with ∼2.6 million residents. Communities served by Downstate experience a greater burden of social and structural determinants of health relative to New York City as a whole. In most of these communities, 90% of the residents are Black or Hispanic, and the life expectancy is ∼9 years less than more affluent communities in New York City.^25^ Approximately one quarter of households live in poverty and 29% experience food insecurity.^25^ The rates of unemployment and being uninsured are higher (8.5% and 11%) compared to New York City (5.9% and 9.8%). These social determinants are inextricably linked to higher rates of disparities such as those observed in chronic kidney disease. The age-adjusted chronic kidney disease hospitalization rates are higher in the borough of Brooklyn compared to New York State overall.^26^ Therefore, addressing disparities related to kidney disease was deemed a priority area for our community.

CERCA was composed of representatives from hospitals and health systems within New York City. Each system was charged with bringing together an internal team composed of multiple key interested parties that would work collaboratively to raise awareness about the use of race corrections, identify a clinical algorithm(s) for intervention, and evaluate the impact of their action. The NYC-DOHMH provided guidance for each hospital through regularly scheduled meetings, providing resources, and offered teams with a network of national leaders with expertise relevant to specific clinical algorithms.

## Sample Participants

### Downstate CERCA team

The Downstate CERCA team included department chairs, hospital leaders, researchers, public health leaders, deans, legal departments, and clinical pathologists. Table 1 depicts the participants in CERCA and their roles. Members of the Downstate CERCA team were selected by invitation from Downstate's Chief Medical Officer who suggested that institutional representatives lend their expertise to achieving the goals of CERCA. Team members were selected based on their expertise in health disparities research, diversity, equity and inclusion, and anti-racism, their racial and ethnic diversity, self-identities, lived experiences, and various academic ranks within the institution. While students were not involved on the team, during this time, students made recommendations to the school leadership expressing the need for the medical schools in general to take a more active stance in advancing equity in clinical practice, and removing race-based practices was among some of their recommendations. A series of steps ensued, including selecting an algorithm to address, conducting key informant interviews, reviewing relevant literature, collecting outcomes data, and reviewing implications with the legal department.

**Table 1.** Representatives in Coalition to End Racism in Clinical Algorithms and Their Roles

Chief Medical Officer who served as the liaison to the larger CERCA group.
Chair of the Department of Medicine who is a nephrologist; provided data on the clinical implication of race-based eGFR; and health disparities researcher.
Chair of the Department of Obstetrics and Gynecology who provided feedback on the use of race in VBAC equation which is not used at Downstate and advanced the understanding of anti-racism in practice; and is health disparities researcher.
Chair of the Department of Pathology and the Director, Clinical Laboratories who provided guidance on process of implementing a new algorithm in clinical practice and the additional resources that were needed to accomplish this change.
Dean of the School of Public Health who provided guidance on population and public health implications.
University General Council raised the importance of identifying established clinical guidelines and considered any legal implications.
Chief for Pulmonary Critical Care who provided feedback on the use of race in assessment of pulmonary function test equation, an algorithm which will be considered for change.
Chief Quality Officer who provided feedback on the association between advancing equity and quality improvement.
Health Disparities Researchers (4) who provided social and scientific guidance on the anti-racism framework and implications for advancing health equity.

CERCA, Coalition to End Racism in Clinical Algorithms; eGFR, estimated glomerular filtration rate; VBAC, vaginal birth after cesarean section.

### Impact

The team met on a weekly basis to identify which clinical algorithm would be selected. A review of the proposed algorithms was conducted with each member describing the rationale for their suggestions. The University Hospital at Downstate did not use VBAC algorithm in clinical care and therefore that was not selected. However, the discussion about VBAC prompted an IRB approved research project focusing on the past use of VBAC in clinical practice. While pulmonary function tests (PFTs) were discussed and deemed worthy of changing, the team felt that more data on alternative algorithms were needed. This discussion has prompted the search for an alternative to race-based PFT and other valid measures. The team arrived at a consensus to tackle eGFR given the disproportionately higher rates of chronic kidney disease (CKD) and associated risk factors observed in the patient population served Downstate and the surrounding community.

We conducted a review of our clinical data and the number of potentially missed patients with CKD. A preliminary study led by Yap et al. found that removing race would reclassify up to 50% of Black patients to higher stages of chronic kidney disease. This finding had implications for pharmacological dosing, making referrals, and outcome surveillance.^27^ The team continued to meet weekly to discuss the process for elimination of race. In general, the team agreed that this was an institutional priority worthy of further investigation. Concerns were raised to ensure that evidence-based approaches were used, which would not adversely impact patient outcomes. The discussions revealed concerns about the clinical impact of removing race for patient outcomes, implications for workload and laboratory personnel, the legal consequences, and challenges of integrating revised algorithms in the medical curriculum. There was also discussion on an alternative algorithm that would be used. Overall, the team was unified in their decision to remove race from eGFR and replace it with a race agnostic, evidence-based, valid, and feasible measure.

This decision was communicated to the director of clinical pathology, and legal consideration of removing race was reviewed by the legal team. Shortly after this Yap et al.,^21^ used our hospital data and performed a single-center, longitudinal retrospective study on a cohort of outpatient African American (AA) patients using the MDRD and MDRD_race removed_ and CKD-EPI and CKD-EPI_race removed_ and their progression to CKD G5 (eGFR <15 mL/min/1.73 m^2^). A total of 327 outpatients were analyzed with a median follow-up of 88.1. When race was removed from MDRD, 39.9% of patients in CKD G1/2 were reclassified to CKD G3a, 72.6% of patients in CKD G3a would be reclassified to CKD G3b, and 54.1% and 36.4% of patients would be reclassified from CKD 3b to CKD G4 and CKD G4 to CKD G5, respectively. Comparing the CKD-EPI formula against the MDRD in our cohort, we found that 8.2%, 18.8%, and 11.4% of patients were reclassified from CKD G1/2 to CKD G3a, CKD G3a to G3b, and CKD G3b to CKD G4, respectively.

Immediately following this publication, new evidence emerged from the CKD Epidemiology Collaboration group using large primary and validation datasets, to develop new eGFR equations without race, now called the CKD-EPI Refit equation.^28,29^ In AA patients, the median decrease in eGFR from CKD-EPI equation using CKD-EPI Refit equation was 3.99 mL/min (range 2.17–5.62). These findings were validated by genetic ancestry data using large datasets from the Chronic Renal Insufficiency Cohort (CRIC) study Investigators to ensure adequacy of race classification. The genetic ancestry data were similar with median eGFR decrease of 3.6 mL/min (range 1.8–5.5) when the CKD-EPI Refit equation was applied in AA.^22,23^

**FIG. 1.**
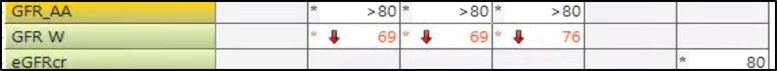
The CKD-EPI formula reported the eGFR by race. The new CKD EPI Refit Equation reports only one value. CKD-EPI, Chronic Kidney Disease Epidemiology Collaboration; eGFR, estimated glomerular filtration rate.

Based on these large studies, the NKF and the ASN endorsed the use of the CKD-EPI Refit equation. The new equation is as follows: CKD-EPIcr_R eGFR=142×min (S_cr_/κ, 1) α×max (S_cr_/κ, 1)^−1.200^ 0.9938^age^×1.012 [if female]. In this equation, S_cr_=standardized serum creatinine in mg/dL, κ=0.7 (females) or 0.9 (males), *α*=−0.241 (female) or −0.302 (male), min (S_cr_/κ, 1) is the minimum of S_cr_/κ or 1.0, and max (S_cr_/κ, 1) is the maximum of S_cr_/κ or 1.0. The Clinical Laboratory accepted this evidence and worked with Information Technology in January of 2022 to incorporate the new formula for eGFR reporting. This new algorithm was updated in computer decision support tools with a ‘go live’ date of February 28, 2022. All laboratory reports were updated to reflect this change. Table 2 provides a timeline for this process. The change was communicated to all clinical staff through an email memo. Figure 1 illustrates the old system of reporting eGFR for AA and White separately and the new eGFR by CKP-EPI Refit as only one value without race classification.

**Table 2.** Time of Coalition to End Racism in Clinical Algorithms Initiatives at Downstate

Date	Initiative
October 4th 2021	Invitation to join CERCA was disseminated by the NYC-DOHMH
Oct 29th 2021	Deadline for Downstate to sign CERCA pledge to join the CERCA initiative
November 22nd 2021	Downstate team had first meeting with representatives from NYC-DOHMH CERCA team
November 2021 to January 2022	Downstate CERCA team met regularly to discuss plans for removing race from eGFR
January 2022 to February 2022	Information technology and clinical laboratory teams met with CERCA teams and legal to finalize plan for updating clinical algorithm and clinical decision support tools.
February 28, 2022	New algorithm was integrated in clinical decision support, all laboratory reports were updated to reflect this change, and this change was disseminated to clinical staff through email memo.

NYC-DOHMH, New York City Department of Health and Mental Hygiene.

The Downstate community response to the CERCA efforts has been positive with institution-wide support among clinical and nonclinical staff. Continued discussions of changes regarding PFTs are ongoing. Retrospective review of prior use of the race based VBAC calculator is in progress in an IRB-based study. Future follow-up by CERCA team members will review next steps and review of community based impact of clinical decisions to patient care. Within the Department of Obstetrics and Gynecology, community engagement will occur through the Downstate's Women's Health Community Advisory Board for bidirectional communication with community members, more specifically regarding the VBAC calculator, which is not in use at our institution.

## Discussion and Health Equity Implications

Removing race from the estimation of glomerular filtration rate is an important start to tackling structural racism in clinical medicine. This initiative provides a model by which a department of health led coalition brought together multiple interested parties to addresses structural racism in clinical algorithms. An important lesson learned in this process was the importance of having support from the local public health department. This provided an external catalyst for change. It brought greater legitimacy to our efforts and provided a wider support base. Having the support of the NYC-DOHMH was critical to this work and allowed us to share best practices with other institutions. The regularly scheduled meetings provided an opportunity to brainstorm and leverage the input of other health systems in addressing challenges.

Another important lesson was the need for institutional level support and support of key leaders in the hospital. This was important to set this as a hospital-wide priority and to encourage clinical faculty to alter their practice. Team members at Downstate included heads of departments, which was crucial to reinforce the importance of this initiative. The multiple sectors involved were important to address both clinical and legal implications. However, while the team implemented this change, there was still a gap in translating the clinical changes to medical education. The medical school curriculum recently began to adapt these changes in clinical didactic sessions. Therefore, it is important to integrate findings and outcomes of CERCA into the medical curriculum and to develop future equity focused clinicians.

CERCA serves as a catalyst and model for other public health-academic partnerships. While this initiative serves as a critical step to reducing disparities in advanced CKD and ultimately end-stage kidney disease, it is equally important that efforts are made to increase diversity in the nephrology workforce and to engage community partners in raising awareness about preventive measures. Future evaluation based on CERCA recommendations include documenting the extent to which this change contributes to differences in prevalence of CKD by stage, rates of referrals made to nephrologists, referrals to dialysis and transplant, and changes in use of potentially nephrotoxic medications. These metrics for evaluation were developed by the NYC CERCA team led by New York City Department of Mental Health and Hygiene.^22^

It is important to note that despite the obvious racist nature of the CKD-EPI formula and the potential benefits of early interventions with removal of race in the formula, concerns were raised to hold off on removing the equation until new evidence was obtained. The primary concern here was patient perception and how patients will react if they were told that their eGFR was lower than previously reported and how to respond if they demanded evidence. The evidence that subsequently became widespread provided a solid foundation for rapid adoption of the CKD EPI Refit equation. Medical societies must follow the good example of the nephrology societies and, where applicable, provide rapid assessments of large datasets to provide validation. Validation will not only provide the scientific premise but also will enhance the policy aspects removal of race in their clinical algorithms.

There are little data on patient perception on inclusion of race in clinical algorithms or in clinical practice. Previous studies have found that most patients are unaware of the incorporation of race in clinical care.^29^ Therefore, the CERCA initiative provides an opportunity to engage patients in these discussions and for developing interventions that incorporate patient perspectives. Involving community members and community organizations to advance this work is also a critical next step.

The CERCA initiative at Downstate has implications for advancing health equity at the level of the health care system. Health equity occurs when everyone has an opportunity to achieve health and no one is disadvantaged based on race or other socially constructed identifiers. From an anti-racism perspective, achieving equity in renal care entails acknowledging that the use of race in eGFR and other clinical algorithms is entrenched in racism and that Black enslaved people were viewed as biologically inferior. These uncomfortable truths in the history of medicine must be acknowledged to correct current practices and ensure that future physicians do not perpetuate the same errors. The second step in anti-racism is making an intentional and concerted effort to dismantle these structures of racism in practice.

The use of race as a biological variable neglects the influence of lived experiences and disregards the notion that observed differences in physiology stem from the embodiment of racism and not race. Using an anti-racism framework and acknowledging the role of racism in clinical practice, our team was able to engage in difficult discussions and collaboratively work to successfully remove the race coefficient from the eGFR. This is an important step toward advancing health equity in clinical practice. Unless we take corrective steps, race becomes codified as a biological construct and its misuse becomes cemented in the practice of medicine.

Addressing structural racism in health care requires team-based approaches and partnerships with key leaders in health such as health departments. CERCA serves as a model for developing academic and public health department partnerships that advance health equity and promote anti-racism in practice. This serves as an example of how the practice and science of community engagement can be enhanced to address policy. Advancing equity requires collection of data through the conduct of research; however, it also calls for academic and community partnerships that tackle systemic racism through policy and practice change. Lessons learned can be adapted to identify, review, and remove the use of race as a coefficient from other clinical guidelines. Studies are needed on patient perceptions of being reclassified into different disease categories when race is removed from clinical algorithms. Such information will help plan implementation strategies.

## Abbreviations Used

AA: African American
ASN: American Society of Nephrology
CERCA: Coalition to End Racism in Clinical Algorithms
CKD-EPI: Chronic Kidney Disease Epidemiology Collaboration
COVID-19: coronavirus disease 2019
CRIC: Chronic Renal Insufficiency Cohort
eGFR: estimated glomerular filtration rate
FRAX: Fracture Risk Assessment Tool
MDRD: Modification of Diet in Renal Disease
NKF: National Kidney Foundation
NYC-DOHMH: New York City Department of Health and Mental Hygiene
PFT: pulmonary function test
SFGH: San Francisco General Hospital
UCSF: University of California-San Francisco
UTI: urinary tract infection
VBAC: vaginal birth after cesarean section
